# Association of different obesity patterns with hypertension in US male adults: a cross-sectional study

**DOI:** 10.1038/s41598-023-37302-x

**Published:** 2023-06-29

**Authors:** Lu Chen, Jun Zhang, Nan Zhou, Jia-Yi Weng, Zheng-Yang Bao, Li-Da Wu

**Affiliations:** 1 Department of Cardiology, The Affiliated Suzhou Hospital of Nanjing Medical University, Suzhou Municipal Hospital, Gusu School, Nanjing Medical University, Suzhou, 215000 China; 2 Department of Internal Medicine, Wuxi Maternity and Child Health Care Hospital, Women’s Hospital of Jiangnan University, Jiangnan University, Wuxi, 214002 China; 3 Health Examination Center, Huadong Sanatorium, Wuxi, 214065 China; 4 Department of Cardiology, Nanjing First Hospital, Nanjing Medical University, Nanjing, 210029 China

**Keywords:** Cardiology, Diseases

## Abstract

Obesity is an important risk factor for hypertension. We aimed to investigate the association between different obesity patterns and hypertension risk in a large male population in the US. Male participants from the National Health and Nutrition Examination Survey (NHANES) (2007–2018) were enrolled in this cross-sectional study. Social demographic information, lifestyle factors, anthropometric measurements and biochemical measurements were collected. Three obesity patterns were classified according to the body mass index (BMI) and waist circumference (WC), including overweight and general obesity, abdominal obesity, and compound obesity. We adopted multivariate logistic regression to investigate the associations between hypertension and different obesity patterns after adjusting for cofounding factors. Subgroup analysis, stratified by age, smoking, drinking and estimated glomerular filtration rate (eGFR), was also conducted to explore the associations between obesity patterns and hypertension risk among different populations. Moreover, the association between WC and hypertension among male individuals was also explored using restricted cubic spline (RCS) analysis. Receiver operating characteristic (ROC) was used to evaluate the discriminatory power of WC for screening hypertension risk. 13,859 male participants from NHANES survey (2007–2018) were enrolled. Comparing with the normal-weight group, the odds ratios (ORs) [95% confidence interval (CI)] for hypertension in individuals with overweight and general obesity, abdominal obesity and compound obesity were 1.41 [1.17–1.70], 1.97 [1.53–2.54] and 3.28 [2.70–3.99], respectively. Subgroup analysis showed that the effect of different obesity patterns on hypertension risk was highly stable among individuals with different clinical conditions. In addition, WC had a positive correlation with the risk of hypertension (OR: 1.43; 95% CI 1.37–1.52; *P* < 0.001) in fully adjusted multivariate logistic regression model. RCS analysis showed that the association between WC and hypertension risk was in a nonlinear pattern, and WC had a good discriminatory power for hypertension in ROC analysis. Different patterns of obesity have a great impact on the risk of hypertension among male individuals. Increment of WC significantly increased the hypertension risk. More attention should be paid to the prevention of obesity, especially abdominal obesity and compound obesity in male individuals.

## Introduction

Hypertension refers to a clinical syndrome characterized by elevated arterial blood pressure^1^. The prevalence rate of hypertension is increasing year by year, with more than 1.3 billion people suffering from hypertension worldwide at present. One in five adults has hypertension. In addition, the age of onset of hypertension tends to be younger. Hypertension is particularly harmful to the blood vessels, heart, kidneys and brain, and accounts for the largest number of deaths from all diseases. The primary treatment goal for patients with hypertension is to minimize the overall risk of cardiovascular complications and death^2^. Unreasonable life style factors, including excessive sodium salt, low potassium diet, heavy alcohol consumption, excessive intake of saturated fatty acids can raise blood pressure. Smoking and heavy alcohol consumption are also risk factors for hypertension. Moreover, obesity is one of the most important risk factors for hypertension. A better understanding of the relationship between hypertension and obesity is crucial to prevent hypertension^3^.

Obesity has already become a serious public health problem and associated with numerous complications, including hyperlipidemia, hypertension, arteriosclerosis and other chronic diseases^4^. In addition, patients with obesity are more likely to have diabetes, gout and some tumor-related diseases^5–7^. Obesity is due to high fat food intake or small amount of activity, leading to the accumulation of fat in the body. Obesity causes thickening of subcutaneous fat, resulting in increased blood volume, and eventually leading to hypertension and even left ventricular hypertrophy^8^. There are three main patterns of obesity at present, including overweight and general obesity, abdominal obesity and compound obesity^9^. Both of abdominal obesity and hypertension are important components of metabolic syndrome, and their common pathogenesis includes insulin resistance, secondary hyperinsulinemia and inflammatory reaction, which can cause damage to heart, kidney and other organs. The main harm of abdominal obesity is that it can lead to diabetes, hyperlipidemia, hypertension, and visceral damage, which is prone to fatty liver and heart disease. In women, it can also lead to breast and cervical cancer. Waist circumference (WC) often serves as an index for abdominal obesity, and body mass index (BMI) is the most used indicator to evaluate the status of general obesity^10^.

Few studies have examined the level of hypertension risk associated with different patterns of obesity. In addition, current studies believe that the prevalence of hypertension in male population is higher than that in female population, and the influence of obesity on hypertension in male population is greater than that in female population^11^. To the best of our knowledge, we firstly focus on the influence of different obesity patterns on hypertension particularly in male population. We conducted a cross-sectional study to further explore the association between different obesity patterns and the hypertension risk in male individuals. Subgroup analyses were also performed to assess the effect of different obesity patterns on the hypertension risk of in male individuals with different clinical conditions.

## Methods

### Study population

National Health and Nutrition Examination Survey (NHANES) is a research program designed to assess the health and nutrition status of adults and children in the US^12^. A mass of data in NHANES database have been analyzed extensively, which is of great help in finding the etiologies, understanding the epidemiology, and searching for novel biomarkers of different diseases^13–15^. To ensure that the samples were representative, the method of stratified multistage probability sampling was used to screen out participants in NHANES survey. In our study, five continuous cycles of the NHANES (2007–2008, 2009–2010, 2011–2012, 2013–2014, 2015–2016, and 2017–2018) were adopted. Male participants with complete demographic data, standard physical measurements, biochemical indicators, and lifestyle information were included in the present study. The exclusion criteria were as follows: (1) age < 18 or ≥ 80 years, (2) estimated glomerular filtration rate (eGFR) < 60 ml/min/1.73 m^2^, (3) participants without key clinical records, including BMI, WC and blood pressure records. This is an observational study performed according to the STROBE Checklist (https://www.strobe-statement.org/checklists/).

### Anthropometric measurements

Experienced examiners measured weight, height, and WC of participants using standardized techniques and equipment. WC, an index for abdominal obesity, was measured at the superior border of the iliac crests and categorized into quartiles. BMI is an indicator of general obesity. The formula of dividing weight (Kg) by the square of height (m) was adopted to calculate BMI (Kg/m^2^)^16^. Detailed procedures for all anthropometric measurements are available on the NHANES website.

### Different obesity patterns

According to the standards of International Diabetes Federation (IDF), normal weight was defined as 18.5 kg/m^2^ ≤ BMI < 23.9 kg/m^2^; BMI ≥ 24.0 kg/m^2^ was regarded as overweight; general obesity was defined as BMI ≥ 28.0 kg/m^2^ without an abnormal WC; abdominal obesity was defined as WC ≥ 102 cm for men and WC ≥ 80 cm for women, with normal BMI at the same time; the coexistence of both general and abdominal obesity was regarded as compound obesity^17^.

### Definition of hypertension

According to the protocol of blood pressure measurement released by the American Heart Association, the blood pressure was recorded by a trained examiner. The average systolic/diastolic blood pressure of three consecutive measurements was obtained and reported. It is the same as the previous published researches on the analysis of NHANES database, hypertension was defined as (1) average SBP ≥ 140 mmHg, (2) average DBP ≥ 90 mmHg, (3) self-reported hypertension; (4) individuals with prescribed antihypertensive medications. The criteria of 140/90 mmHg refers to the guideline of International Society of Hypertension^18^.

### Covariates

We selected covariates according to the previously published studies^19–21^. Age, race/ethnicity, and education level were obtained from the demographic questionnaire. Diabetes history, alcohol consumption and smoking status were adopted from the health questionnaire. After at least 8 h of an overnight fast, blood samples were collected and used to examine the levels of triglyceride (TG), total cholesterol (TC), low-density lipoprotein cholesterol (LDL-C), high-density lipoprotein cholesterol (HDL-C), red blood cell (RBC), white blood cell (WBC), blood platelet (PLT), neutrophil (NE), lymphocyte (LY), hemoglobin, HemoglobinA1c (HbA1c), fast blood glucose (FBG), aspartate aminotransferase (ALT) and aspartate transaminase (AST). NHANES website provided the detailed procedures in collecting biochemical measurements.

### Statistical analysis

Continuous variables were presented as the mean ± standard deviation (SD) (normal distribution), or the median (interquartile range) (skewed distribution). We adopted Kolmogorov–Smirnov test to assess the normality. Categorical variables were presented as the number (percentage). We compared baseline characteristics among individuals with different obesity patterns based on Chi-square test for categorical variables and one-way ANOVA test (normal distribution) or Shapiro–Wilk test (skewed distribution) for continuous variables. Kernel density estimation was used to illustrate distributions of WC in individuals with and without hypertension. To evaluate the associations between different obesity patterns and WC with hypertension risk, multiple logistic regression analysis was performed after adjusting for confounding factors selected by the stepwise backward selection method (age, race/ethnicity, education, smoking, drinking, diabetes, TG, TC, LDL-C, HDL-C, RBC, hemoglobin and eGFR). Individuals with normal-weight were the reference and the odds ratios (ORs) with 95% confidence intervals (CIs) were calculated. Considering overweight is a risk factor for hypertension, the overweight and general obesity populations were merged into a single group as previous studies. Subgroup analyses stratified by age, smoking, drinking and eGFR were performed to further assess the associations between different obesity patterns and hypertension. Restricted cubic spline analysis (RCS) (with three piecewise points) was used to evaluate the nonlinear associations between WC and the risk of hypertension, the median value of WC was used as a reference. Receiver operating characteristic (ROC) curve was used to evaluate the discriminative power of WC in identifying individuals with hypertension. A *P* value < 0.05 was considered significant. All statistical analyses were conducted using R software (R Core Team, 2022; version 4.1.6) and SPSS 25.0 software (SPSS Institute, Chicago).

### Ethics approval and consent to participate

The new ethic statement as follows: The NCHS Ethics Review Board protects the rights and welfare of NHANES participants. The NHANES protocol complies with the U.S. Department of Health and Human Services Policy for the Protection of Human Research Subjects. NCHS IRB/ERC Protocol number: 2011–17. Ethical review and approval were waived for this study as it solely used publicly available data for research and publication. Informed consent was obtained from all subjects involved in the NHANES. This study was deemed exempt from review by the Ethics Committee of Huadong Sanatorium.

## Results

### Characteristics of the study population

**Figure 1:**
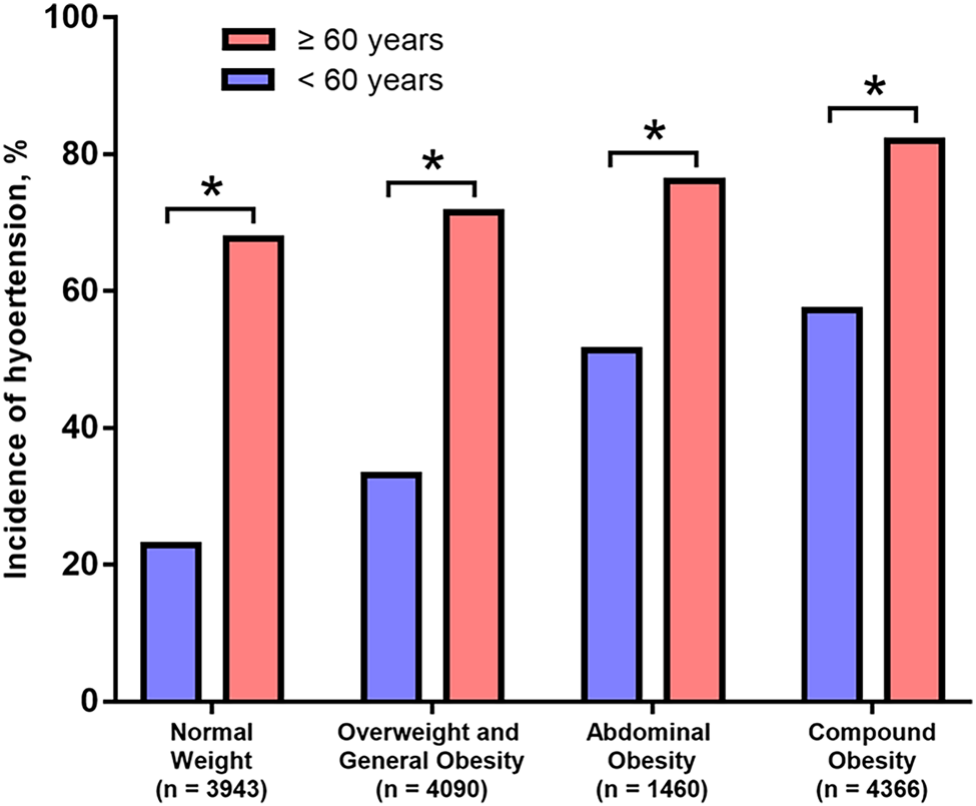
Comparison of the prevalence rate of hypertension between individuals ≥ 60 years and individuals < 60 years among different obesity patterns (n = 13,859).

13,859 male participants from NHANES (2007–2018) was enrolled in the present study. The proportions of compound obesity, abdominal obesity and overweight and general obesity were 4,366 (31.5%), 1,460 (10.5%) and 4090 (29.5%), respectively. The prevalence of hypertension among compound obesity, abdominal obesity, overweight and general obesity and normal weight groups were 2800 (64.1%), 916 (62.7%), 1643 (40.2%) and 1269 (32.0%). A significant difference was observed in the prevalence of hypertension among age ≥ 60 years group and age < 60 years in the normal weight, overweight and general obesity, abdominal obesity and compound obesity groups (p < 0.05) (Fig. 1). Significant differences in demographic characteristics, blood biochemical indexes and life style factors were observed among the different obesity patterns (Table 1).

**Table 1:** Comparison of patients’ characteristics among different obesity patterns (n = 13,859).

Variables	Normal weight (n = 3943)	Overweight and general obesity (n = 4090)	Abdominal obesity (n = 1460)	Compound Obesity (n = 4366)	*P* values
Age, years	38 (24, 56)	41 (30, 54)	58 (46, 67)	48 (35, 61)	< 0.001
WC, cm	84.0 (78.7, 89.3)	95.6 (91.9, 98.9)	105.2 (103.3, 107.9)	115.5 (109.1, 124.2)	< 0.001
BMI, kg/m^2^	22.8 (21.3, 23.9)	27.1 (26.0, 28.5)	28.6 (27.5, 29.7)	34.1 (31.8, 37.6)	< 0.001
SBP, mmHg	118.3 (110.1, 128.7)	113.1 (106.2, 130.9)	125.3 (116.6, 135.3)	125.1 (116.9, 136.2)	< 0.001
DBP, mmHg	70.2 (62.3, 77.1)	72.1 (65.6, 79.8)	73.6 (65.2, 80.3)	75.1 (67.2, 83.1)	< 0.001
Race/ethnicity, %					< 0.001
Non-Hispanic White	1457 (37.0%)	1272 (31.1%)	785 (53.8%)	1851 (42.4%)	
Non-Hispanic Black	930 (23.6%)	871 (21.3%)	206 (14.1%)	996 (22.8%)	
Mexican American	440 (11.2%)	834 (20.4%)	206 (14.1%)	816 (18.7%)	
Other Hispanic	300 (7.6%)	525 (12.8%)	153 (10.5%)	416 (9.5%)	
Other races	816 (20.7%)	588 (14.4%)	110 (7.5%)	287 (6.6%)	
Education levels, %					< 0.001
Below high school	311 (7.9%)	459 (11.2%)	165 (11.3%)	384 (8.8%)	
High school	1712 (43.5%)	1578 (38.6%)	566 (38.8%)	1766 (40.5%)	
Above high school	1916 (48.6%)	2051 (50.2%)	728 (49.9%)	2212 (50.7%)	
Diabetes, %	309 (7.8%)	474 (11.6%)	341 (23.4%)	1178 (27.0%)	< 0.001
Smoking, %	1946 (52.4%)	1879 (47.6%)	899 (61.9%)	2226 (51.7%)	< 0.01
Drinking, %	1318 (49.4%)	1508 (51.7%)	458 (44.6%)	1538 (50.1%)	< 0.001
eGFR, ml/min/1.73m^2^	103.6 (89.7, 117.5)	99.0 (86.3, 111.9)	90.4 (78.2, 101.6)	96.8 (83.5, 110.5)	< 0.001
TG, mmol/L	0.94 (0.67, 1.37)	1.19 (0.82, 1.73)	1.24 (0.93, 1.88)	1.41 (0.99, 2.01)	< 0.001
TC, mmol/L	4.63 (3.98, 5.30)	4.89 (4.19, 5.56)	4.89 (4.19, 5.60)	4.86 (4.16, 5.56)	< 0.001
LDL-C, mmol/L	2.66 (2.17, 3.28)	2.97 (2.41, 3.60)	2.92 (2.38, 3.57)	2.97 (2.38, 3.62)	< 0.001
HDL-C, mmol/L	1.37 (1.16, 1.63)	1.22 (1.03, 1.42)	1.19 (1.01, 1.40)	1.11 (0.96, 1.29)	< 0.001
RBC, × 10^9^/L	4.90 (4.62, 5.17)	4.99 (4.73, 5.26)	4.91 (4.62, 5.18)	5.02 (4.74, 5.28)	< 0.001
WBC, × 10^9^/L	6.42 (5.31, 7.82)	6.63 (5.61, 8.03)	7.12 (5.93, 8.40)	7.42 (6.11, 8.82)	< 0.001
PLT, × 10^6^/L	22.3 (19.2, 25.8)	22.6 (19.5, 26.3)	22.3 (18.8, 25.9)	23.0 (19.5, 26.7)	< 0.001
NE, × 10^9^/L	3.62 (2.81, 4.73)	3.71 (2.92, 4.71)	4.10 (3.22, 5.12)	4.31 (3.43, 5.46)	< 0.001
LY, × 10^9^/L	1.92 (1.63, 2.42)	2.03 (1.72, 2.61)	2.02 (1.62, 2.41)	2.13 (1.69, 2.56)	< 0.001
Hemoglobin, g/L	15.0 (14.3, 15.7)	15.1 (14.5, 15.9)	15.0 (14.3, 15.9)	15.1 (14.4, 15.9)	< 0.001
HbA1c, %	5.4 (5.1, 5.7)	5.5 (5.2, 5.8)	5.6 (5.3, 6.0)	6.2 (5.7, 7.4)	< 0.001
FBG, mmol/L	5.4 (5.1, 5.9)	5.6 (5.3, 6.1)	5.8 (5.4, 6.5)	5.9 (5.5, 6.8)	< 0.001
ALT, u/L	20.0 (16.0, 27.0)	25.0 (20.0, 34.0)	24.0 (19.0, 33.0)	28.0 (22.0, 39.0)	< 0.001
AST, u/L	23.0 (20.0, 28.0)	25.0 (21.0, 30.0)	24.0 (21.0, 30.0)	24.0 (21.0, 29.0)	< 0.001

Normally distributed continuous variables are presented as the mean ± standard deviation; Non-normally distributed continuous variables are presented as the mean (interquartile range); Categorical variables are presented as the number (percentage).

*WC* waist circumference, *BMI* body mass index, *SBP* systolic blood pressure, *DBP* diastolic blood pressure, *eGFR* estimated glomerular filtration rate, *TG* triglycerides, *TC* total cholesterol, *LDL-C* low-density lipoprotein cholesterol, *HDL-c* high-density lipoprotein cholesterol, *RBC* red blood cells, *WBC* white blood cells, *PLT* platelets, *NE* neutrophils, *LY* lymphocytes, *HbA1c* glycated hemoglobin, *FBG* fasting blood glucose, *ALT* alanine aminotransferase, *AST* glutamic transaminase.

### Association between different obesity patterns and hypertension

In the non-adjusted model, compound obesity (OR, 3.81; 95% CI, 3.48–4.17,* P* < 0.001), abdominal obesity (OR, 3.59; 95% CI, 3.16–4.07, *P* < 0.001) and overweight and general obesity (OR, 1.43; 95% CI, 1.31–1.57, *P* < 0.001) were all strongly associated with hypertension risk. In the minimally adjusted model, after adjusting for confounding factors, the associations between different obesity patterns and hypertension risk decreased. However, all of these three obesity patterns were still independent factors associated with increased hypertension risk. After adjusting for age, race/ethnicity, education, smoking, drinking, diabetes, TG, TC, LDL-C, HDL-C, RBC, hemoglobin and eGFR, individuals in the compound obesity group had more than three-fold increase in the odds of developing hypertension (OR: 3.28; 95% CI 2.70–3.99; *P* < 0.001) compared with individuals with normal weight in the fully adjusted model. People with abdominal obesity were nearly twice as likely of developing hypertension (OR:1.97, 95% CI 1.53–2.54; *P* < 0.001). Overweight and general obesity also associated with the hypertension risk (OR:1.41, 95% CI 1.17–1.70; *P* < 0.001) (Table 2).

**Table 2:** Multivariate logistic regression model of associations between different patterns of obesity and hypertension Risk (n = 13,859).

Obesity Patterns	Total	Hypertension (n, %)	Non-adjusted model		Minimally adjusted model		Fully adjusted model	
			OR [95% CI]	*P*-value	OR [95% CI]	*P*-value	OR [95% CI]	*P*-value
Normal weight	3943	1260 (32.0%)	Reference	–	Reference	–	Reference	–
Overweight and general obesity	4090	1643 (40.2%)	1.43 [1.31, 1.57]	*P* < 0.001	1.37 [1.21, 1.54]	*P* < 0.001	1.41 [1.17, 1.70]	*P* < 0.001
Abdominal obesity	1460	916 (62.7%)	3.59 [3.16, 4.07]	*P* < 0.001	1.86 [1.58, 2.19]	*P* < 0.001	1.97 [1.53, 2.54]	*P* < 0.001
Compound obesity	4366	2800 (64.1%)	3.81 [3.48, 4.17]	*P* < 0.001	3.23 [2.86, 3.64]	*P* < 0.001	3.28 [2.70, 3.99]	*P* < 0.001

Minimally adjusted model, we adjusted for age, race/ethnicity, education, smoking, drinking. Fully adjusted model, we adjusted for age, race/ethnicity, education, smoking, drinking, diabetes, TG, TC, LDL-C, HDL-C, RBC, hemoglobin, and eGFR.

*TG* triglycerides, *TC* total cholesterol, *LDL-C* low-density lipoprotein cholesterol, *HDL-C* high-density lipoprotein cholesterol, *RBC* red blood cells, *eGFR* estimated glomerular filtration rate.

### Subgroup analysis of different obesity patterns and the risk of hypertension

**Figure 2:**
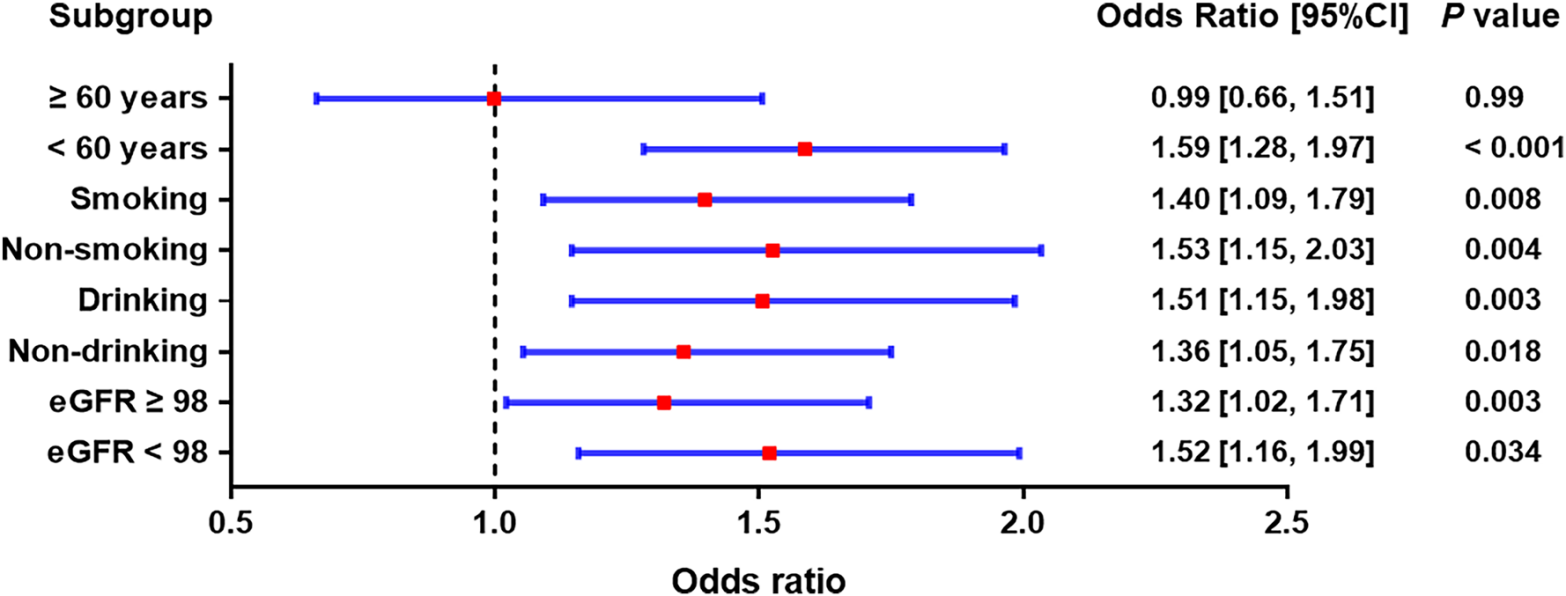
Subgroup analyses for the risks of developing hypertension in the overweight and general obesity group compared with the normal-weight group.

**Figure 3:**
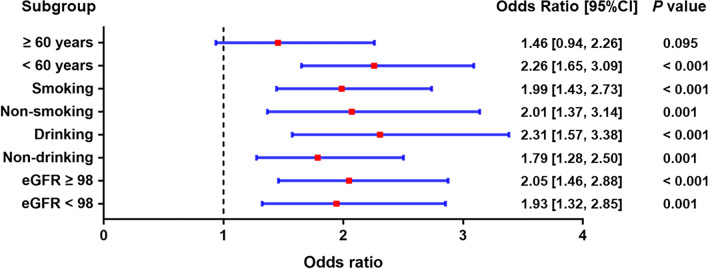
Subgroup analyses for the risks of developing hypertension in the abdominal obesity group compared with the normal-weight group.

**Figure 4:**
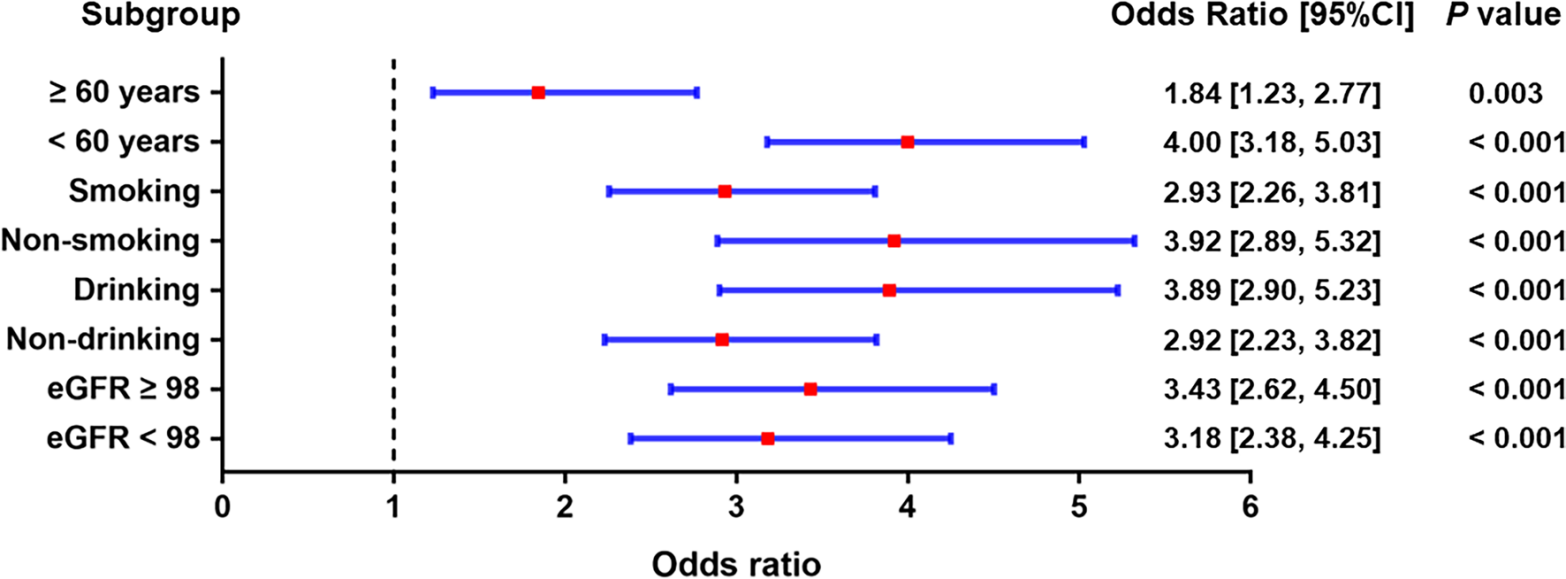
Subgroup analyses for the risks of developing hypertension in the compound obesity group compared with the normal-weight group.

We also carried out subgroup analyses stratified by age, smoking, drinking and eGFR, after adjusting for age, race/ethnicity, education, smoking, drinking, diabetes, TG, TC, LDL-C, HDL-C, RBC, hemoglobin, and eGFR. In Figs. 2, 3, 4, compared with normal weight at baseline, the risk of hypertension was found to increase in all three obesity patterns. Our results showed that the effects of different obesity patterns on hypertension risk was highly stable. In each subgroup, individuals with compound obesity were all associated with a high-risk value of developing hypertension (Fig. 3). Nevertheless, abdominal obesity and overweight and general obesity were not associated with the increased prevalence rate of hypertension in the population of age more than 60 years group (Figs. 2, 3). We also found that, across subgroups, the risk of hypertension was increased in individuals with overweight and general obesity than with normal weight, but higher in individuals with abdominal obesity, individuals with compound obesity having the highest risk.

### Association between WC and hypertension

**Figure 5:**
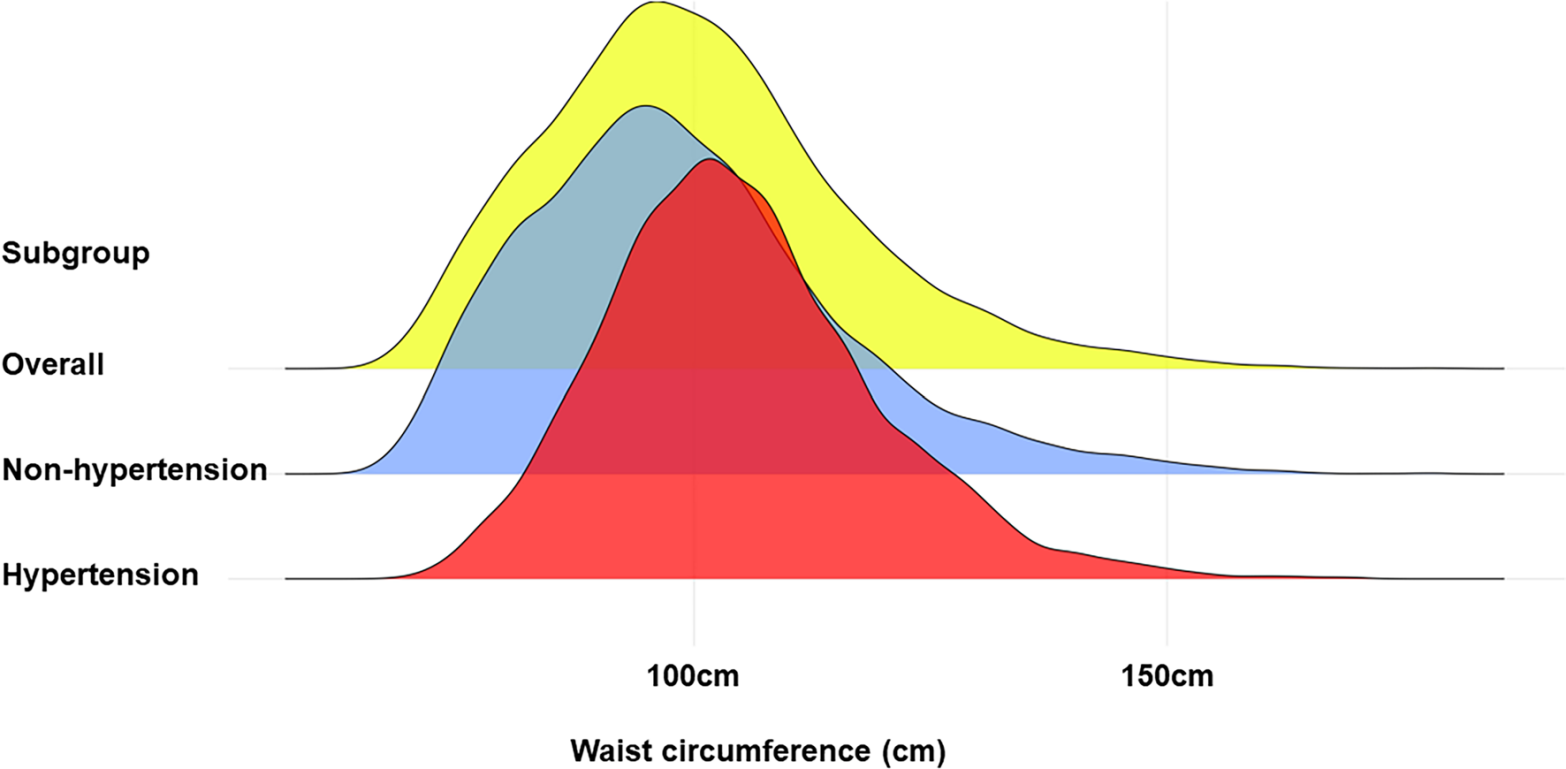
The overall distribution of WC and distribution of WC in individuals with hypertension and individuals without hypertension.

**Figure 6:**
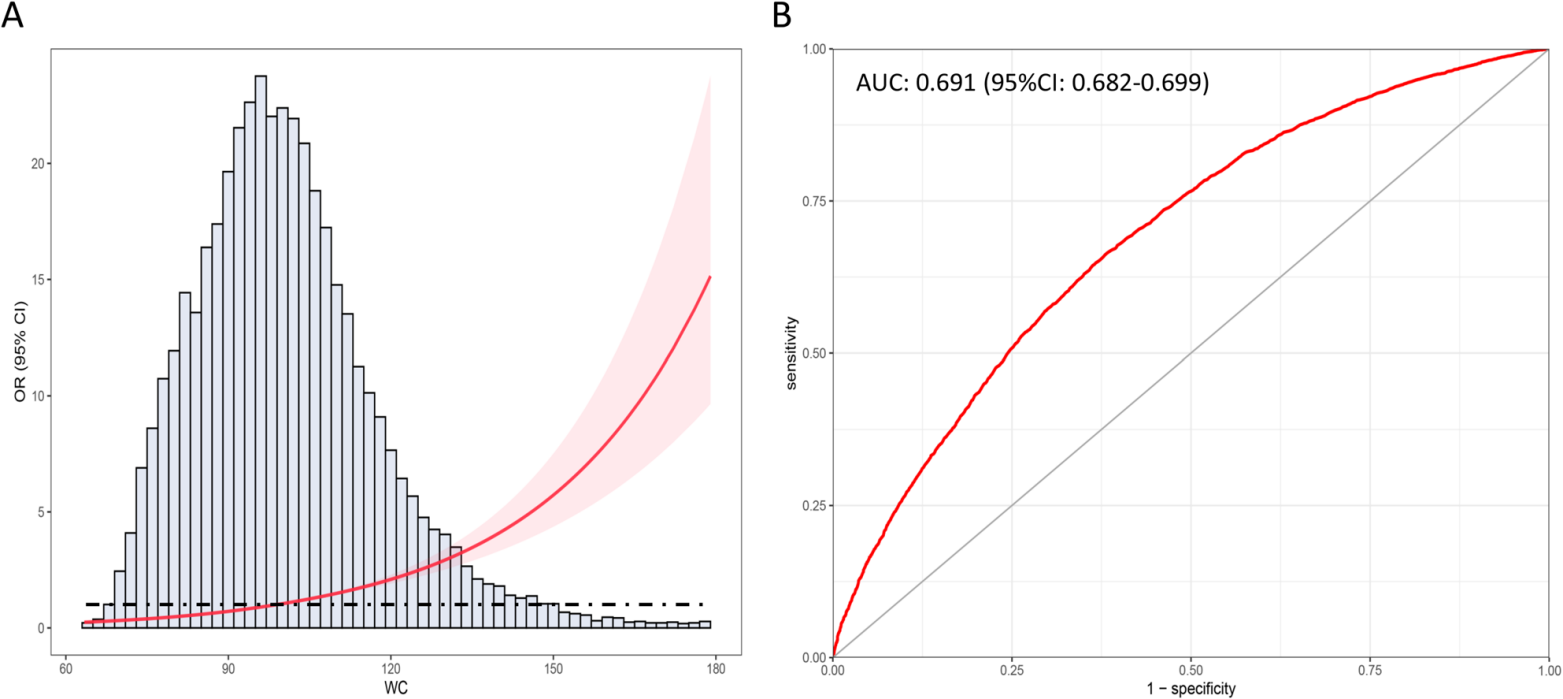
RCS analysis between WC and the risk of hypertension and the ROC curve. (**A**) RCS analysis for the association between WC and the risk of hypertension; (**B**) ROC curve of WC for discriminating hypertension risk.

Considering individuals with abdominal obesity and compound obesity had a larger WC and a significantly higher prevalence rate of hypertension, we explored the relationship between WC and hypertension. The distribution of WC stratified by hypertension is provided in Figs. 5. Increased WC (per 10 cm) had a positive correlation with the prevalence rate of hypertension in non-adjusted model (OR: 1.58; 95% CI 1.54–1.62; *P* < 0.001), minimally adjusted model (OR: 1.42; 95% CI 1.38–1.47; *P* < 0.001) and fully adjusted model (OR: 1.43; 95% CI 1.37–1.52; *P* < 0.001) (Table 3). When fully adjusting for age, race/ethnicity, education, smoking, drinking, diabetes, TG, TC, LDL-C, HDL-C, RBC, hemoglobin and eGFR, individuals in the second to the fourth quartile of WC still had a higher risk for hypertension. Results of RCS analysis also demonstrated that WC were positively corelated with the increased prevalence rate of hypertension, and in a nonlinear pattern. The hypertension risk increased rapidly with the increase of WC, especial in the upper quantile (Fig. 6A). ROC analysis showed that WC had a well discriminatory power for screening hypertension risk, and the AUC of WC is 0.691 (95% CI 0.682–0.699; optimal cutoff value: 98.2) (Fig. 6B).

**Table 3:** Multivariate logistic regression model of associations between waist circumference and hypertension risk (n = 13,859).

Obesity Patterns	Total	Hypertension (n, %)	Non-adjusted model		Minimally adjusted model		Fully adjusted model	
			OR [95% CI]	*P*-value	OR [95% CI]	*P*-value	OR [95% CI]	*P*-value
Waist circumference (per 10 cm)	13,859	6619 (47.8%)	1.58 [1.54, 1.62]	*P* < 0.001	1.42 [1.38, 1.47]	*P* < 0.001	1.43 [1.37, 1.52]	*P* < 0.001
Categories								
Q1	3482	883 (25.4%)	Reference	–	Reference	–	Reference	–
Q2	3459	1462 (42.3%)	2.16 [1.95, 2.39]	*P* < 0.001	1.40 [1.23, 1.60]	*P* < 0.001	1.51 [1.23, 1.87]	*P* < 0.001
Q3	3459	1924 (55.6%)	3.69 [3.33, 4.08]	*P* < 0.001	2.18 [1.91, 2.49]	*P* < 0.001	2.37 [1.91, 2.95]	*P* < 0.001
Q4	3459	2350 (67.9%)	6.24 [5.62, 6.92]	*P* < 0.001	3.92 [3.42, 4.50]	*P* < 0.001	4.10 [3.26, 5.16]	*P* < 0.001

Minimally adjusted model, we adjusted for age, race/ethnicity, education, smoking, drinking. Fully adjusted model, we adjusted for age, race/ethnicity, education, smoking, drinking, diabetes, TG, TC, LDL-C, HDL-C, RBC, Hemoglobin, and eGFR.

*TG* triglycerides, *TC* total cholesterol, *LDL-C* low-density lipoprotein cholesterol, *HDL-C* high-density lipoprotein cholesterol, *RBC* red blood cells, *eGFR* estimated glomerular filtration rate.

## Discussion

In the present cross-sectional study, we included 13, 859 male participants from NHANES (2007–2018) to explore the association between the hypertension risk and different obesity patterns in male population. BMI and WC were used as indicators for general obesity and abdominal obesity, respectively. The prevalence rate of hypertension among male individuals with compound obesity (64.1%), abdominal obesity (62.7%), overweight and general obesity (40.2%) and normal weight (32.0%) was evaluated in detail. Moreover, after adjusting for covariates, we carried out multivariable regression analysis to further investigate the increased risk of hypertension in male individuals with different obesity patterns compared with individuals with normal weight. Results of multivariable regression analysis showed that different obesity patterns were independent risk factors for hypertension in male population. The risk of hypertension in individuals with abdominal obesity and compound obesity was higher than individuals with overweight and general obesity. Therefore, different obesity patterns have a great impact on the prevalence of hypertension in male population. Multivariable regression analysis was also performed to investigate the association between WC and hypertension, which showed that increased WC was also an independent risk factor for hypertension^22^.

Back in the 1960s, the Framingham heart study found that obesity significantly increased the risk of hypertension^23^. In recent years, the prevalence of hypertension and obesity has increased significantly worldwide^24^. The research on the pathogenesis of obesity-related hypertension is helpful to find out reasonable treatment methods. Obesity can be mainly divided into overweight and general obesity, abdominal obesity and compound obesity. Many studies have explored the effects of different obesity patterns on different diseases. Lu et al. performed a retrospective study and found that different types of obesity had significant effects on the risk of Hashimoto's thyroiditis, and compound obesity was an independent risk factor^17^. Barcelar et al. also demonstrated that obesity, particularly the abdominal obesity, was associated with respiratory system alterations, and severe abdominal obesity could eventually lead to respiratory dysfunction^25^. In addition, abdominal obesity is also closely associated with nonalcoholic fatty liver disease, diabetes, coronary heart disease and other chronic diseases^26^. In the present cross-sectional study, we firstly investigated the effects of different obesity patterns on the risk of hypertension based on data from NHANES database. Our results showed that obesity patterns had significant effects on the prevalence rate of hypertension in male population. Male population are more prone to metabolic disorders, which may explain why different obesity patterns had a greater impact on the prevalence of hypertension in male population. In addition, we also conducted a detailed subgroup analysis stratified by age, smoking, drinking, and eGFR, the effect of different obesity patterns on hypertension was highly stable. Results of subgroup analysis demonstrated that, among individuals older than 60 years, only compound obesity was the independent factor. This may be due to a significant increase in the prevalence rate of hypertension due to the hardening of blood vessels with increasing age.

Using multivariable regression analysis, we found that the prevalence of hypertension was significantly higher in individuals with either type of obesity patterns than individuals with normal weight. However, individuals with abdominal obesity or compound obesity had a higher risk of hypertension than individuals with overweight and general obesity. Abdominal obesity is a kind of abnormal phenotypes of fat distribution, and is associated with insulin resistance and chronic inflammation, which can lead to hypertension through multiple mechanisms^27^. In abdominal obesity, the levels of RAAS components such as renin, angiotensin, and aldosterone are significantly increased, and the elevated levels of aldosterone exceed the renin activity. In addition to renal RAAS activation, RAAS of cardiac system, RAAS of vascular system, RAAS of adipose system and RAAS of central nervous system are also significantly activated, eventually leading to hypertension^28^. Vascular dysfunction in abdominal obesity includes changes in vascular structure, endothelial dysfunction and increased vascular stiffness. Abnormal endothelial function and increased vascular stiffness are the main changes in the early stage of obesity-induced hypertension, and insulin resistance is an important mechanism to initiate this process^29^. Increased body volume load and impaired renal function are important clinical features of obesity. In recent years, researchers found that impaired renal function may occur before obesity-induced hypertension happens^30^. The activation of sympathetic nervous system caused by obesity is also an important mechanism of obesity related hypertension. Moreover, leptin levels are elevated in most individuals with obesity, suggesting leptin resistance. Hyperleptinemia can promote norepinephrine conversion and increase sympathetic activity, leading to elevated blood pressure^31^.

Most studies used BMI as the main criterion for assessing obesity at present, however, BMI does not accurately reflect the distribution of body fat^32^. WC is one of the most commonly used indicators of anthropometry and widely used for the diagnosis of abdominal obesity. According to the guidelines recommended by the IDF, it is necessary to combine BMI and WC in the assessment of obesity, which is helpful for the relationship between different forms of obesity and different diseases^33^. Several studies have already investigated associations between WC and clinical outcomes in patients with type 2 diabetes^34^. A recent prospective case–control study examined the associations between circulating levels of inflammation factors and demonstrated that WC is associated with an increased coronary heart disease (CHD) risk independent of other underlying risk factors such as physical activity levels and BMI^35,36^. Another systematic review and meta-analysis suggest that regular aerobic exercise can moderately reduce WC and visceral adipose tissue accumulation, and reduce the risk of cardiovascular and cerebrovascular diseases, while high-intensity exercise may benefit patients without underlying chronic diseases more than moderate intensity exercise^37^. We also investigated the exact relationship between WC and the hypertension risk, and our results also showed that increased WC could be an independent risk factor for hypertension. Elevated WC can lead to the secretion of interleukin (IL) -6 and tumor necrosis factor (TNF) -α from white adipose cells in the body, leading to the infiltration of macrophages, and eventually chronic inflammation^38^. The function of infiltrated islet cells is affected and insulin resistance eventually occurs. On the other hand, inflammatory factors can affect the normal secretion and regulation function of vascular endothelial cells, resulting in the loss of normal function of endothelial cells^39^. Besides, we found that the association between WC and hypertension was in a nonlinear pattern, the risk of hypertension increased significantly with the increment of WC, especially in the upper quantile. Controlling of WC and abdominal obesity is very important for the control of hypertension^40^. Moreover, WC can be also adopted to screen out the risk of hypertension in male individuals.

Although we conducted a detailed analysis of the effects of different obesity patterns on the prevalence of hypertension, there are still some limitations to be noted in this study. First, this study used only a nationally representative sample from the US, but there are large ethnic differences in diet, physical activity, genetic variants, lipid metabolism, and susceptibility to cardiovascular disease, and the generality of our conclusions to other populations is unclear. Second, due to the inherent nature of cross-sectional studies, it is difficult to determine the causal relationship between different modes of obesity and hypertension^41^. More prospective studies are needed to determine the exact relationship between different forms of obesity and hypertension. Third, although we adjusted for multiple covariates, we could not completely exclude the influence of other confounding factors on our results.

## Conclusions

In conclusion, we investigated the association of different obesity patterns with hypertension and found that abdominal obesity and compound obesity were strongly associated with increased risk of hypertension. With the increasing burden of hypertension and its resulting cardiovascular and cerebrovascular diseases worldwide, the management of obesity, especially abdominal obesity, should be strengthened to prevent hypertension.

## Supplementary Information

Supplementary Table S1.

## Data Availability

Publicly available datasets were analyzed in this study. All the raw data used in this study are derived from the public NHANES data portal (https://wwwn.cdc.gov/nchs/nhanes/analyticguidelines.aspx).
