# Supplementary material for: Association of different obesity patterns with hypertension in US male adults: a cross-sectional study
Source: Sci Rep. 2023 Jun 29;13:10551. doi: 10.1038/s41598-023-37302-x (PMC10310720; doi:10.1038/s41598-023-37302-x)
Supplement: Supplementary file 1 — Supplementary Table S1. [file 41598_2023_37302_MOESM1_ESM.docx]

**Table S1 Comparison of Patients’ Characteristics Among Patients with Hypertension and without Hypertension (n = 13859)**

| Variables | Overall  (n = 13859) | Hypertension  (n = 6619) | Non-hypertension  (n = 7240) | *P* values |
| --- | --- | --- | --- | --- |
| Age, years | 44 (31, 59) | 54 (40, 63) | 36 (27, 50) | < 0.001 |
| WC, cm | 98.0 (89.6, 109.0) | 104.0 (95.0, 114.8) | 94.5 (85.7, 103.5) | < 0.001 |
| BMI, kg/m^2^ | 27.7 (24.5, 31.5) | 29.1 (25.9, 33.5) | 26.6 (23.6, 29.8) | < 0.001 |
| SBP, mmHg | 121.0 (113.2, 131.6) | 132.0 (122.3, 142.6) | 115.6 (109.2, 121.1) | < 0.001 |
| DBP, mmHg | 72.3 (65.1, 79.3) | 77.6 (68.2, 85.7) | 69.1 (63.6, 75.1) | < 0.001 |
| Race/ethnicity, % |  |  |  |  |
| Non-Hispanic White | 5365 (38.7%) | 2589 (39.1%) | 2776 (38.3%) |  |
| Non-Hispanic Black | 3003 (21.7%) | 1714 (25.9%) | 1289 (17.8%) |  |
| Mexican American | 2296 (16.6%) | 954 (14.4%) | 1342 (18.5%) |  |
| Other Hispanic | 1394 (10.1%) | 618 (9.3%) | 776 (10.7%) |  |
| Other races | 1801 (13.0%) | 744 (11.2%) | 1057 (14.6%) |  |
| Education levels, % |  |  |  |  |
| Below high school | 1319 (9.5%) | 165 (11.3%) | 384 (8.8%) |  |
| High school | 5622 (40.6%) | 566 (38.8%) | 1766 (40.5%) |  |
| Above high school | 6907 (49.9%) | 728 (49.9%) | 2212 (50.7%) |  |
| Diabetes, % | 2302 (16.6%) | 341 (23.4%) | 1178 (27.0%) | < 0.001 |
| Smoking, % | 6950 (51.8%) | 899 (61.9%) | 2226 (51.7%) | < 0.001 |
| Drinking, % | 4822 (49.8%) | 458 (44.6%) | 1538 (50.1%) | < 0.001 |
| eGFR, ml/min/1.73m^2^ | 99.1 (86.2, 111.8) | 93.9 (80.95, 106.3) | 103.7 (91.0, 116.3) | < 0.001 |
| TG, mmol/L | 1.19 (0.82, 1.74) | 1.28 (0.88, 1.86) | 1.12 (0.77, 1.63) | < 0.001 |
| TC, mmol/L | 4.86 (4.19, 5.53) | 4.91 (4.22, 5.61) | 4.81 (4.16, 5.48) | < 0.001 |
| LDL-C, mmol/L | 2.92 (2.35, 3.54) | 2.92 (2.34, 3.57) | 2.97 (2.35, 3.52) |  |
| HDL-C, mmol/L | 1.24 (1.03, 1.47) | 1.19 (1.01, 1.40) | 1.11 (0.96, 1.29) | < 0.001 |
| RBC, ×10^9^/L | 4.99 (4.73, 5.26) | 4.97 (4.69, 5.26) | 5.02 (4.78, 5.25) | < 0.001 |
| WBC, ×10^9^/L | 6.40 (5.40, 7.70) | 6.60 (5.50, 7.90) | 6.30 (5.30, 7.50) | < 0.001 |
| PLT, ×10^6^/L | 22.3 (19.2, 25.9) | 22.2 (19.0, 25.9) | 22.3 (19.3, 25.9) |  |
| NE, ×10^9^/L | 3.62 (2.81, 4.63) | 3.81 (3.02, 4.75) | 3.52 (2.73, 4.41) | < 0.001 |
| LY, ×10^9^/L | 1.92 (1.63, 2.42) | 1.91 (1.53, 2.32) | 2.03 (1.62, 2.41) | 0.046 |
| Hemoglobin, g/L | 15.2 (14.5, 15.9) | 15.2 (14.4, 15.9) | 15.3 (14.6, 15.9) | < 0.001 |
| HbA1c, % | 5.5 (5.2, 5.8) | 5.6 (5.3, 6.0) | 5.4 (5.2, 5.6) | < 0.001 |
| FBG, mmol/L | 5.3 (5.7, 6.2) | 5.9 (5.4, 6.6) | 5.6 (5.2, 5.9) | < 0.001 |
| ALT, u/L | 25.0 (19.0, 34.0) | 25.0 (20.0, 36.0) | 24.0 (19.0, 32.0) | < 0.001 |
| AST, u/L | 24.0 (21.0, 29.0) | 25.0 (21.0, 30.0) | 24.0 (20.0, 28.0) | < 0.001 |

Normally distributed continuous variables are presented as the mean ± standard deviation; Non-normally distributed continuous variables are presented as the mean (interquartile range); Categorical variables are presented as the number (percentage). WC, waist circumference; BMI, body mass index; SBP, systolic blood pressure; DBP, diastolic blood pressure; eGFR, estimated glomerular filtration rate; TG, triglycerides; TC, total cholesterol; LDL-C, low-density lipoprotein cholesterol; HDL-c, high-density lipoprotein cholesterol; RBC, red blood cells; WBC, white blood cells; PLT, platelets; NE, neutrophils; LY, lymphocytes; HbA1c, glycated hemoglobin; FBG, fasting blood glucose; ALT, alanine aminotransferase; AST, glutamic transaminase.
